# Clonal and Plasmid-Mediated Dissemination of Multidrug-Resistant *Salmonella* Enteritidis in Chicken Production, Northeastern Thailand

**DOI:** 10.3390/pathogens15010075

**Published:** 2026-01-10

**Authors:** Zhihui Zhang, Fanan Suksawat, Xue Zhang, Xianghua Shu, Sunpetch Angkititrakul

**Affiliations:** 1 Faculty of Veterinary Medicine, Khon Kaen University, Khon Kaen 40000, Thailand; zhihui.z@kkumail.com (Z.Z.); sjirap@kku.ac.th (F.S.); 2 College of Veterinary Medicine, Yunnan Agricultural University, Kunming 650201, China; xuezhangx@outlook.com

**Keywords:** *Salmonella*, antimicrobial resistance, plasmids, One Health, mobile genetic elements, northeastern Thailand

## Abstract

Background: The global dissemination of multidrug-resistant (MDR) *Salmonella* poses a persistent and serious threat to food safety systems. As a leading poultry-exporting country, Thailand requires a comprehensive understanding of how resistance plasmids spread among *Salmonella* populations within its chicken production chain. Methods: Between March 2023 and February 2024, 223 *Salmonella* isolates were collected from chicken slaughterhouses and markets in northeastern Thailand. From these, 19 representative MDR *Salmonella enterica* isolates, selected based on distinct spatiotemporal distributions, underwent whole-genome sequencing. Genomic analyses included sequence typing, core-genome phylogenetics, and screening for antimicrobial resistance genes. Plasmid replicons were identified, and functional annotation was performed using the COG database. Results: Phylogenetic analysis revealed 11 distinct sequence types within the population. Among these, ST1541 and ST50 showed clear evidence of clonal transmission across different production stages, with a notable clustering pattern observed during the winter season. All sequenced isolates exhibited an MDR phenotype. Plasmids were detected in 78.9% of isolates, with conjugative plasmids being the most frequent type (57.9%). The β-lactamase gene *bla*_TEM-60_ was the most prevalent (78.9%) and showed a strong correlation (r ≥ 0.7) with resistance to both ampicillin and cefotaxime. Functional annotation further revealed an abundance of genes involved in carbohydrate and amino acid metabolism across all isolates. Conclusions: These findings indicate that MDR *Salmonella* dissemination is driven by two synergistic mechanisms: the clonal expansion of fit lineages and the horizontal transfer of conjugative plasmids harboring β-lactamase genes. We identified IncI-gamma-K1 and Col-related plasmids as key vectors in this process. This study advocates for targeted interventions, guided by a One Health approach, that specifically aim to disrupt plasmid transmission at critical control points, such as slaughterhouses, to curb the spread of antimicrobial resistance.

## 1. Introduction

*Salmonella* infections remain a major public health concern, causing a significant annual burden of gastroenteritis and invasive diseases [1]. Poultry, especially chicken, serves as a primary reservoir in commercial production and contributes to frequent foodborne outbreaks and substantial economic losses worldwide [2]. Among non-typhoidal *Salmonella* serovars, *Salmonella enterica* serovar Enteritidis (hereafter *S. enteritidis*) is of paramount importance. It is a common foodborne, facultative intracellular enteropathogen [3]. Surveillance data consistently highlight its high prevalence in poultry-associated human infections [4]. This is particularly evident in Thailand and Southeast Asia, where *S. enteritidis* is frequently reported as one of the predominant serovars, accounting for approximately 43.0% of human salmonellosis cases [5]. The persistence of this serovar in human populations is underpinned by its endemicity in poultry reservoirs. In Thailand, *Salmonella* contamination remains a key challenge in chicken production, with studies from slaughterhouses reporting an average prevalence of 34.8% in chicken carcasses, where *S. enteritidis* is a commonly isolated serotype [6]. Retail markets serve as critical endpoints and potential amplifiers of this contamination; for instance, a study in Thai-Cambodian border provinces reported a *Salmonella* prevalence of 65.7% in retail chicken meat [7]. This high level of contamination at the point of sale facilitates the transmission of pathogens to consumers [8] and underscores the successful adaptation of *S. enteritidis* to the poultry production chain and its significant role in the regional foodborne disease burden.

Notably, the epidemiology of *S. enteritidis* is dynamic, with transmission dynamics influenced by spatiotemporal factors. For example, contamination levels vary according to the location within the production chain. *S. enteritidis* represents a considerable proportion of contaminants in retail chicken meat [9]. Importantly, studies suggest a potential trend of contamination dispersal from slaughterhouses to retail markets, largely attributable to post-processing handling and hygiene practices [10,11]. This indicates that contamination at retail is not merely a static endpoint but may reflect an amplification of upstream transmission dynamics. Furthermore, seasonal climatic conditions may affect its prevalence. In tropical regions like Thailand, the warm and humid rainy season creates an environment conducive to bacterial survival and spread [12]. *Salmonella enterica* demonstrates remarkable adaptability to commercial poultry production environments [13]. It maintains persistent colonization in slaughterhouses and retail markets, which serve as critical amplification points for community-wide dissemination [14]. These factors continue to pose ongoing challenges for food safety and public health.

The use of antimicrobial agents in food animal production creates selective pressure that favors the emergence and spread of multidrug-resistant (MDR) *Salmonella*, defined as non-susceptibility to three or more antimicrobial classes [15]. Slaughterhouses and retail markets harbor dense microbial communities, providing ideal conditions for gene exchange. In these environments, mobile genetic elements (MGEs), a class of DNA fragments capable of moving within or between genomes, including plasmids, transposons, and integrons, facilitate the horizontal transfer of antimicrobial resistance genes (ARGs) across species barriers [16,17]. The acquisition of ARGs through MGEs, combined with the clonal expansion of well-adapted bacterial strains, forms a dual mechanism driving the persistence and dissemination of MDR *Salmonella* within integrated food production networks, a phenomenon particularly evidenced in Southeast Asia [18,19]. This gene exchange allows pathogenic strains to acquire resistance-conferring clusters from environmental and commensal bacteria, thereby significantly exacerbating public health concerns.

Despite substantial evidence indicating a relatively high prevalence of MDR *Salmonella* in Thailand’s chicken production [20,21], critical knowledge gaps remain. Specifically, the spatiotemporal dynamics of MDR strain transmission are still unclear, and the precise epidemiological roles of prevalent plasmids have yet to be elucidated. Genome studies across Asia have revealed distinct regional patterns: certain plasmid families, particularly the prevalent IncI1 and IncFIB types, are consistently associated with MDR *Salmonella* in poultry environments. These plasmids often carry extended-spectrum β-lactamase (ESBL) genes, including clinically significant *bla*_CTX-M_ variants [22,23]. Meanwhile, specific clonal lineages such as *S. enteritidis* ST11 and *S. infantis* ST32 have emerged as dominant vehicles for resistance plasmids across multiple Asian production systems. While these studies highlight significant interactions between successful clones and MGEs, the exact compositional characteristics, transmission dynamics, and seasonal fluctuations of these high-risk genetic combinations within Thailand’s chicken production network remain poorly characterized. A detailed understanding of these factors is essential for developing effective intervention strategies.

Therefore, this study applied whole-genome sequencing (WGS) to delineate the clonal relationships, resistance gene profiles, and plasmid mobility patterns of MDR *Salmonella* circulating within the Thai chicken production system. This approach not only generated descriptive data but also enabled an in-depth investigation of the functional transmission mechanisms driving the persistence of MDR *Salmonella*. By analyzing *S*. Enteritidis isolates from different seasons and locations, this study, for the first time, establishes high-resolution, spatiotemporally defined genomic profiles of antimicrobial resistance (AMR) transmission. These findings provide critical insights for prevention and control strategies, supporting the One Health framework’s requirements for monitoring AMR and identifying key control points within the chicken production chain.

## 2. Materials and Methods

### 2.1. Bacterial Isolates and Study Design

From March 2023 to February 2024, continuous monitoring of *Salmonella* was conducted across the chicken production chain in northeastern Thailand. Sampling occurred over three distinct seasons: summer, rainy, and winter. A total of 689 samples were collected from both broiler chicken slaughterhouses and retail markets. The slaughterhouses operated under standardized protocols with established biosecurity measures, while the open-air retail markets maintained basic visual cleanliness and were subject to routine official inspections but did not implement additional protective measures. Samples consisted of cloacal swabs from chickens and surface swabs from carcasses, collected at both slaughterhouse and market locations. *Salmonella* isolation strictly adhered to the ISO standard [24]. Briefly, samples were pre-enriched in buffered peptone water, followed by transfer to a semi-solid medium and selective enrichment in modified semi-solid Rappaport-Vassiliadis medium. Isolates were then streaked onto xylose lysine deoxycholate agar for colony selection. Presumptive *Salmonella* colonies were confirmed using biochemical tests. This procedure yielded 223 *Salmonella*-positive isolates. Serotyping was performed according to the White-Kauffmann-Le Minor scheme via slide agglutination with commercial antisera (Denka Seiken, Tokyo, Japan). Following serotyping, all 223 isolates were tested for antimicrobial susceptibility against 14 antibiotics using the disk diffusion method (OXOID, Liofilchem, UK) on Mueller-Hinton agar (Difco Laboratories, Detroit, MI, USA), following CLSI guidelines [25]. *Escherichia coli* ATCC 25,922 served as the quality control strain. Among the isolates, 25 were identified as *S. enteritidis*. To capture spatiotemporal and resistance diversity within the constraints of sequencing capacity, a subset of 19 representative *S. enteritidis* isolates was selected for WGS. Selection criteria ensured that all isolates exhibited an MDR phenotype and represented all three seasons (summer, *n* = 3; rainy, *n* = 6; winter, *n* = 10) and both sampling locations (slaughterhouse, *n* = 9; market, *n* = 10). All isolates were stored as glycerol stocks at −80 °C in a regional reference laboratory for long-term preservation and future studies. All experiments were conducted in a standard laboratory setting. Due to the limited number of sequenced strains, the findings provide supporting data and a reference for the monitored period and specific production chain but do not constitute a comprehensive epidemiological survey of *S. enteritidis* across wider geographical areas.

### 2.2. DNA Extraction and WGS

Genomic DNA was extracted from overnight bacterial cultures using the Takara MiniBEST Bacteria Genomic DNA Extraction Kit (Takara Bio Inc., Shiga, Japan) following the manufacturer’s instructions. DNA concentration and quality were assessed using a Qubit fluorometer (Thermo Fisher Scientific, Waltham, MA, USA) and agarose gel electrophoresis. Electrophoresis showed that the extracted DNA was of high molecular weight, indicating intact, high-quality DNA suitable for WGS and capable of producing long reads. WGS was performed on the Illumina NovaSeq 6000 platform (Illumina (San Diego, CA, USA) BGI (Shenzhen, China)), generating 2 × 150 bp paired-end reads.

### 2.3. Genomic Analysis Pipeline

Raw sequencing reads were quality-trimmed using Trimmomatic v0.39 [26] and subsequently assembled into contigs with SPAdes v3.11.1 [27]. The quality of the genome assemblies was evaluated using QUAST v5.0.2 [28], while CheckM v1.0.11 assessed genome completeness and detected potential contamination [29]. In silico serotyping and multilocus sequence typing (MLST) were performed using mlst v2.11 (https://github.com/tseemann/mlst, accessed on 16 October 2025). ARGs and plasmid replicons were identified using ABRicate v1.0.1 with the comprehensive antibiotic resistance database (CARD) [30] and the PlasmidFinder database [31] respectively, applying minimum thresholds of 90% coverage and 95% identity. The genetic context of plasmid contigs was characterized, typed, and reconstructed using Mob-suite v3.1.9 [32]. Protein sequences were functionally annotated with the EggNOG-mapper tool against the Clusters of Orthologous Groups (COG) database, and virulence factors were identified using the virulence factors database (http://www.mgc.ac.cn/VFs/, accessed on 16 October 2025).

Genotype-Phenotype Concordance Analysis: The agreement between the presence of acquired AMR genes and the corresponding phenotypic resistance was evaluated for each isolate-antibiotic combination. Results for each combination were classified as follows: True positive (TP): The relevant AMR gene was detected, and the isolate exhibited phenotypic resistance. False negative (FN): The relevant AMR gene was detected, but the isolate was phenotypically susceptible. False positive (FP): The relevant AMR gene was not detected, but the isolate exhibited phenotypic resistance. The overall genotype-phenotype concordance was calculated using the formula: Concordance (%) = [TP/(TP + FN + FP)] × 100%.

### 2.4. Phylogenetic Analysis

Core genome single-nucleotide polymorphisms (SNPs) were identified using the Snippy pipeline v4.6.0 (https://github.com/tseemann/snippy, accessed on 16 October 2025), with the complete genome sequence of *S. enteritidis* P125109 serving as the reference. This strain was chosen for its high-quality, complete genome and its widespread use in *Salmonella* comparative genomic studies. The GenBank assembly accession number is GCF_000009505.1, with nucleotide accession numbers NC_011294.1 (chromosome) and NC_011295.1 (large virulence plasmid). A multiple sequence alignment of the core SNPs was generated and used to infer phylogenetic relationships. The maximum-likelihood phylogenetic tree was constructed using TreeBest v1.9.2 (https://github.com/Ensembl/treebest, accessed on 16 October 2025) with default parameters. The resulting tree was visualized and annotated using the ggtree package in R Studio version 4.5.2. Additionally, Spearman rank correlation analysis and hierarchical cluster analysis were performed to further investigate genetic relationships.

## 3. Results

### 3.1. Genome Assembly and Quality Assessment

High-quality draft genomes were successfully assembled for all 19 *S*. Enteritidis isolates (Table 1). The assemblies demonstrated excellent completeness, with 15 isolates (78.9%) achieving 100% completeness and the remaining four ranging from 99.71% to 99.97%. Assembly contiguity was strong, as reflected by N50 values between 183,935 bp and 528,820 bp. Most genomes were assembled into a relatively low number of contigs (40–96), except for isolate C84A, which comprised 334 contigs. All assembled sequences were gapless. Genomic features were highly consistent across the collection, with genome sizes ranging from 4.67 to 5.09 Mbp and GC contents between 51.70% and 52.25%. The high quality and completeness of these assemblies provide a robust foundation for downstream phylogenetic analyses and detailed investigations of AMR and virulence determinants.

### 3.2. Functional Annotation of Coding Sequences

**Figure 1:**
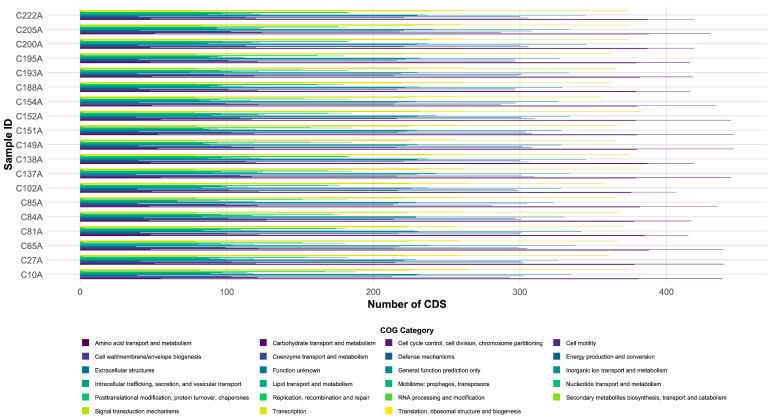
Functional gene categorization of *Salmonella* isolates. Distribution of coding sequences (CDS) across clusters of orthologous groups (COG) functional categories for the 19 *Salmonella* isolates.

To characterize the functional potential of the 19 *Salmonella* isolates, their coding sequences were categorized using the clusters of orthologous groups (COG) database (Figure 1). The largest proportion of coding sequences across all isolates were associated with carbohydrate and amino acid transport and metabolism, indicating robust metabolic adaptability. Notably, the mobilome category exhibited the greatest inter-strain variability (66–132 CDSs), reflecting active horizontal gene transfer. In contrast, categories directly related to AMR and virulence, including cell wall/membrane/envelope biogenesis (281–310 CDSs), defense mechanisms (98–113 CDSs), inorganic ion transport and metabolism (228–243 CDSs), and signal transduction mechanisms (220–239 CDSs), were consistently represented across all isolates. Core cellular functions such as translation and transcription showed minimal variation, demonstrating evolutionary conservation of essential processes. This contrasts with the high diversity observed in MGEs. The resulting genomic architecture, which combines stable core functions with dynamic accessory genes, supports both population stability and adaptive potential. Such a configuration likely contributes to the success of dominant MDR clones under antimicrobial pressure.

### 3.3. Genomic Features of Salmonella

**Figure 2:**
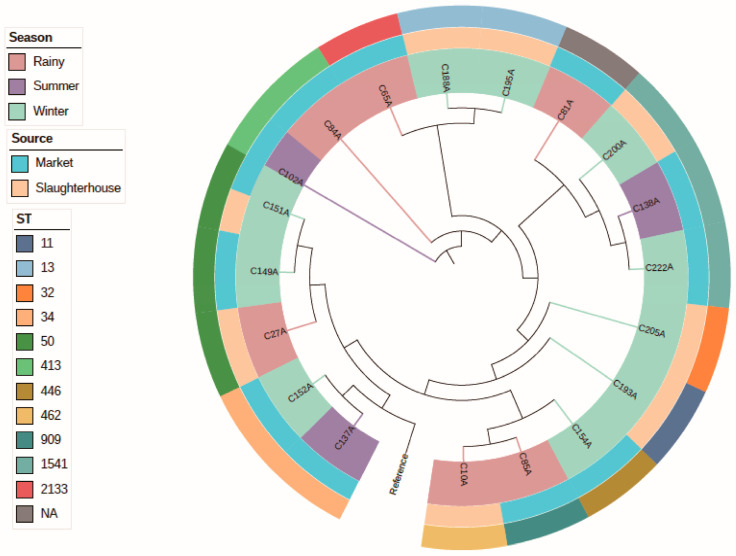
Phylogenetic tree of 19 *Salmonella* isolates based on core genome single-nucleotide polymorphisms (SNPs), colored by season and sampling source. This evolutionary tree integrates MLST sequence data, ST typing, seasonality, and source information. Branches represent genetic relationships, with the first outer ring indicating the season of isolation and the second ring denoting sample sources (circles for slaughterhouses and markets). The outermost ring labels display isolates numbers and STs. Collectively, it reveals the distribution patterns of isolates across temporal, spatial, and genotypic dimensions.

Core genome SNP-based phylogenetic analysis resolved the population structure into distinct clonal clusters, predominantly defined by sequence types (STs) and exhibiting spatiotemporal patterns (Figure 2). Isolates sharing identical STs formed robust monophyletic clusters, such as ST1541 (C138A, C200A, C222A) and ST50 (C27A, C149A, C151A), which were isolated from both slaughterhouse and market environments, indicating successful transmission across production stages. A clear phylogenetic pattern correlated with seasonality: winter isolates formed a tight cluster, whereas isolates from the rainy season were dispersed across multiple branches. This pattern suggests a potential association between season and bacterial population dynamics within the production chain, although further studies are needed to confirm this relationship.

### 3.4. ARGs Profile

**Figure 3:**
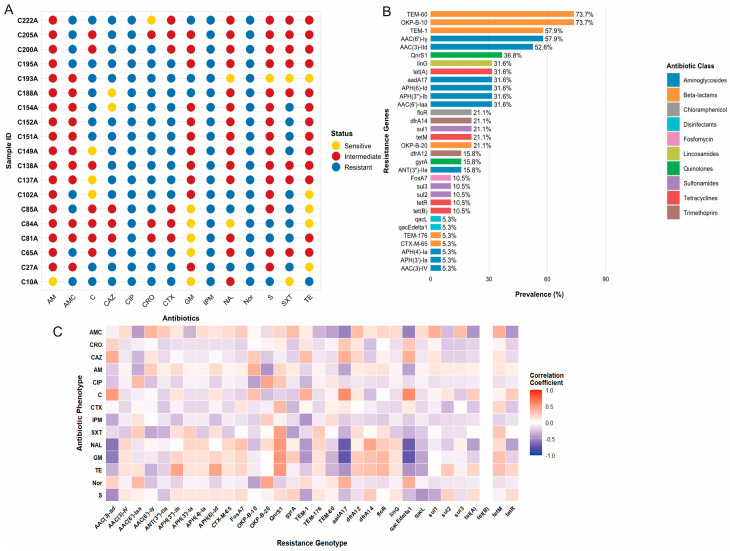
AMR profiles of *Salmonella* isolates. (**A**) Heatmap of phenotypic susceptibility profiles against 14 antibiotics. Blue, susceptible (S); yellow, intermediate (I); red, resistant (R). Antibiotics and disk concentrations: streptomycin (S, 10 μg), norfloxacin (NOR, 10 μg), tetracycline (TE, 30 μg), gentamicin (GM, 10 μg), sulfamethoxazole/trimethoprim (SXT, 23.75 μg), imipenem (IPM, 10 μg), cefotaxime (CRO, 30 μg), chloramphenicol (C, 30 μg), ciprofloxacin (CIP, 5 μg), nalidixic acid (NA, 30 μg), ampicillin (AM, 10 μg), ceftazidime (CAZ, 30 μg), ceftriaxone (CTX, 30 μg), and amoxicillin/clavulanic acid (AMC, 20/10 μg). (**B**) Prevalence of antimicrobial resistance genes (ARGs) in *Salmonella* isolates (**C**). Heatmap of Spearman correlation coefficients between phenotypic resistance and genetic determinants. The color gradient indicates the correlation strength, ranging from −1 (blue) to +1 (red).

All isolates were phenotypically confirmed as MDR, with 68.4% (13/19) resistant to three or more antimicrobial classes. While all isolates remained susceptible to norfloxacin, imipenem, and ciprofloxacin, resistance was frequently observed against amoxicillin-clavulanic acid (47.4%), tetracycline (42.1%), and cefotaxime (26.3%) (Figure 3A). Genomic analysis identified a diverse resistome dominated by β-lactamase genes, with *bla*_TEM-60_ (78.9%), *bla*_OKP-B-10_ (73.7%), and *bla*_TEM-1_ (68.4%) being the most prevalent (Figure 3B). Correlation analysis revealed strong positive associations (Spearman’s r ≥ 0.7) between these β-lactamase genes and resistance to ampicillin and cefotaxime. Hierarchical clustering based on these correlations segregated isolates into three distinct groups. Cluster I comprised MDR isolates from both slaughterhouse and market environments that concurrently carried β-lactamase, tetracycline, and sulfonamide resistance genes, representing a high-risk subpopulation with co-located resistance determinants (Figure 3C).

**Figure 4:**
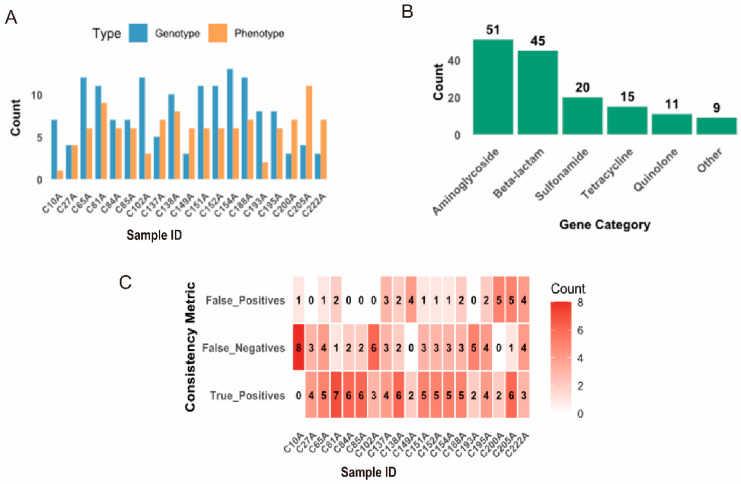
Comparison of genotypic and phenotypic AMR profiles. (**A**) Number of acquired AMR genes (blue bars) versus number of antibiotics with phenotypic resistance (orange bars) for each isolate. (**B**) Proportional distribution of antimicrobial resistance (AMR) gene categories across the 19 isolates. (**C**) The heatmap depicts the count for each consistency metric using a uniform color gradient, with intensity increasing from white (low) to dark red (high). The metrics are distinguished by their row position: True Positives (top row), False Negatives (middle row), and False Positives (bottom row).

To evaluate the consistency between genetic determinants and observed resistance, this study examined genotype-phenotype relationships in AMR (Figure 4). Comparing the number of acquired AMR genes with phenotypic resistance profiles revealed considerable variation among isolates (Figure 4A). On average, isolates carried 7.95 ARGs and exhibited resistance to six antibiotics. β-lactam resistance genes were the most prevalent, followed by aminoglycoside and tetracycline resistance genes (Figure 4B). Across all isolate-antibiotic combinations, genotype-phenotype concordance was 61.7% (Figure 4C). However, this agreement varied widely between isolates, ranging from 100% in isolate C149A to 0% in isolate C10A, suggesting the presence of additional uncharacterized resistance mechanisms or regulatory factors in certain isolates.

### 3.5. MGEs and S. Enteritidis Pathogenic Island (SPI) Profile

**Figure 5:**
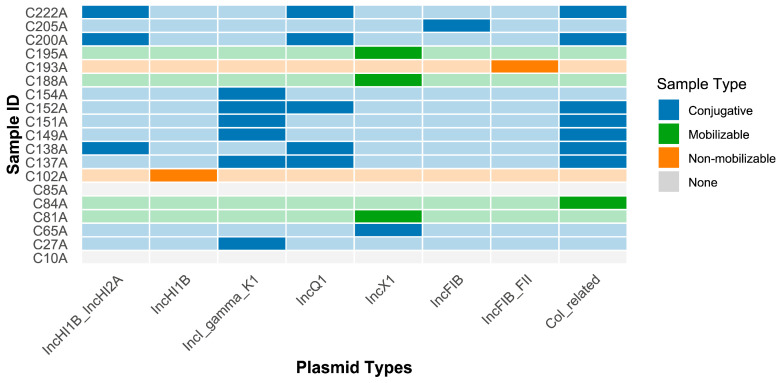
Samples on the y-axis are color-coded according to their predominant plasmid mobility type: blue represents isolates carrying conjugative plasmids, green represents those with mobilizable plasmids, orange denotes isolates harboring non-mobilizable plasmids, and gray indicates isolates where no plasmid was detected. For each plasmid column, its designated color hue represents the presence of that replicon in a sample, while background/blank (or white) cells within the same column indicate its absence (different shades of color).

Plasmid analysis revealed a high plasmid burden, with 78.9% (15/19) of isolates carrying at least one plasmid, and conjugative plasmids being the predominant type (57.9%, 11/19) (Figure 5). The IncI-gamma-K1 and Col-related replicons were the most prevalent, detected in 8 and 9 isolates, respectively. A key finding was the specific association between plasmids and ARGs, highlighting major vehicles for resistance transmission. A perfect co-occurrence was observed between the IncI-gamma-K1 plasmid and the *bla_TEM-60_* gene, present in all eight IncI-gamma-K1-positive isolates (see Figure 5 and Supplementary Table S1). Furthermore, four isolates (C137A, C138A, C200A, C222A) simultaneously carried multiple conjugative plasmids, representing hotspots for ARG accumulation with an average of 10.5 resistance genes, significantly above the study-wide average of 7.95. Another notable finding was the absolute linkage of the IncX1 replicon with the *qnrS1* gene (4/4), identifying IncX1 as a dedicated vector for plasmid-mediated quinolone resistance (PMQR) in this population.

**Figure 6:**
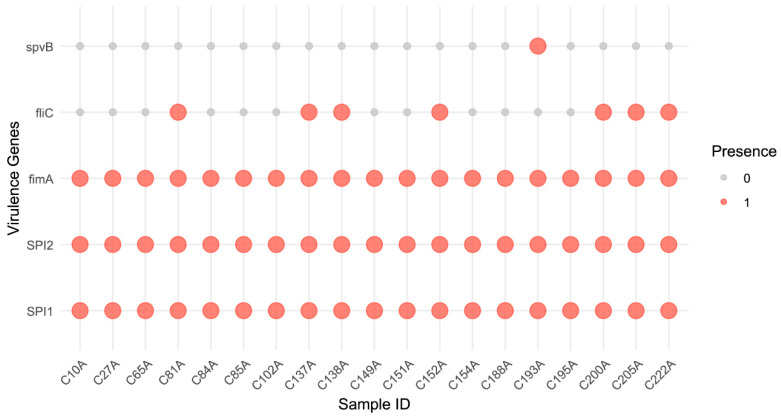
Key virulence gene profiles of *Salmonella* isolates. Heatmap of key virulence genes. Gene presence is indicated in red; absence is indicated in gray.

Virulence gene analysis revealed distinct patterns among the *Salmonella* isolates (Figure 6). All 19 isolates harbored the core SPI-1 and SPI-2 type III secretion system genes, indicating conserved mechanisms for invasion and intracellular survival. The flagellin gene *fliC* was detected in 36.8% (7/19) of isolates, while the plasmid-borne *spvB* gene, associated with systemic infection, was present in only one isolate (C193A, 5.3%). Based on combinations of virulence genes, three distinct profiles emerged: the majority of isolates (12/19) carried only SPI-1 and SPI-2; six isolates possessed SPI-1, SPI-2, and *fliC*; and one isolate (C193A) contained SPI-1, SPI-2, and *spvB*. This distribution suggests varying pathogenic potentials, with the *spvB*-positive isolate representing a high-risk clone due to its enhanced capability for systemic dissemination. To further elucidate the complex interrelationships between plasmid types and resistance genes, a co-occurrence network analysis was performed. The resulting network (Supplementary Figure S1) revealed four major functional clusters, with plasmids such as IncI1_1_Alpha, IncX1, and IncQ1 serving as core hubs, underscoring their pivotal role in connecting and disseminating antimicrobial resistance determinants within this ecosystem.

## 4. Discussion

This study provides new insights into the spatiotemporal distribution of *S. enteritidis* within the chicken production chain in northeastern Thailand. Our genomic data indicate that the dissemination of MDR *Salmonella* in this system is driven by two key processes: clonal expansion of well-adapted lineages and plasmid-mediated horizontal gene transfer. Together, these mechanisms create a resilient network of AMR that challenges conventional control strategies. The detection of identical STs (ST1541 and ST50) in both slaughterhouses and retail markets highlights the persistent circulation of successful clonal lineages, likely maintained by genetic adaptation and competitive fitness. Simultaneously, the widespread presence of conjugative plasmids, particularly IncI-gamma-K1 and Col-related types, facilitates efficient horizontal transfer of resistance genes across bacterial species, enabling resistance spread independently of clonal expansion. These findings provide strong data support for ongoing surveillance efforts and underscore the need for targeted interventions to disrupt both clonal transmission and plasmid-mediated resistance within the poultry production chain.

Core-genome SNP-based phylogenetic analysis confirmed ST1541 and ST50 as the dominant clones within the local chicken production chain, demonstrating transmission pathways between slaughterhouses and markets. This finding should be considered within the broader context of *Salmonella* epidemiology in Southeast Asia. In Thailand and neighboring regions, certain clonal complexes are closely linked to intensive farming systems. For example, S. Typhimurium ST34, carrying multidrug-resistance plasmids, has emerged as a successful and persistently disseminated clone in regional pig and poultry industries [10,33]. The expansion patterns of ST1541 and ST50 observed in our study resemble those of ST34 and the globally successful *S. enteritidis* ST11 lineage, the latter having achieved dominance in poultry production worldwide through international trade of breeding stocks [20]. However, unlike ST11, which is often characterized by large, stable resistance megaplasmids (e.g., pESI), our dominant clones disseminated resistance via smaller, mobile conjugative plasmids (IncI-gamma-K, Col) [34,35]. This distinction underscores a potentially region-specific AMR ecology where plasmid mobility plays a paramount role. Notably, the association of ST50 (which belongs to the same complex as ST1925) with invasive human infections in Singapore [36] suggests that these poultry-derived clones in our region may also pose a potential zoonotic risk. This sentence presents a reasonable interpretation of our own results—namely, the observed phylogenetic clustering of winter isolates. It proposes a hypothesis that seasonal environmental factors may influence bacterial population dy-namics and transmission. As such, it constitutes part of our discussion and speculation based on the findings of this study, rather than a statement of established fact that requires external citation.

All sequenced isolates in this study exhibited an MDR phenotype, with particularly prominent resistance to β-lactams, primarily mediated by the highly prevalent *bla*_TEM-60_ and *bla_OKP-B-10_* genes. This resistance profile aligns closely with recent national surveillance data on *Salmonella* from poultry in Thailand, which report persistently high resistance rates to ampicillin and third-generation cephalosporins, alongside widespread presence of *TEM*-type β-lactamase genes [6]. However, attributing this resistance pattern solely to antimicrobial use is insufficient; it must also be understood in the context of the ecological characteristics of intensive poultry farming systems in Southeast Asia. These systems are characterized by high stocking densities, multi-batch production, and frequent live bird movement. Although Thailand has banned the use of antimicrobials as growth promoters, historical long-term application at sub-therapeutic doses has exerted significant selective pressure on the environment, while current antimicrobial use for disease prevention and treatment continues to maintain this pressure [37]. This persistent ecological selection has not only favored the fixation of resistance genes such as *bla*_TEM-60_ but also promoted the co-persistence of resistance to tetracyclines (*tet*(A)) and sulfonamides (*sul2*) through genetic co-localization within multidrug-resistance regions on plasmids. Therefore, the local AMR profile reflects a complex interplay of historical practices, current management strategies, and ongoing ecological selection pressures in Thailand’s and Southeast Asia’s poultry farming systems.

Plasmids, particularly conjugative types, were identified as the primary vectors driving the cross-strain dissemination of key resistance genes in this study. The strong association between the IncI-gamma-K1 plasmid and *bla_TEM-60_*, along with the absolute linkage between IncX1 and qnrS1, highlights highly specialized plasmid-gene transmission units. This stable partnership likely results from the synergy between the high conjugative efficiency of IncI plasmids in enteric environments [38] and the strong selective advantage conferred by the *bla_TEM-__sub_* ESBL gene under persistent β-lactam selection pressure in poultry production [39]. This phenomenon is consistent across the region. Studies indicate that IncI family plasmids are major vehicles for spreading ESBL and plasmid-mediated AmpC genes among gut bacteria in livestock throughout Southeast Asia, with evidence of cross-border transmission [40]. Similarly, IncX1 plasmids carrying qnrS1 have been reported in the Malaysian poultry industry, constituting a regional PMQR dissemination network [41]. The mobility of these plasmids facilitates resistance gene transfer across species barriers, allowing movement from poultry production chains into the environment and potentially into human pathogens, exemplifying a classic One Health threat. Moreover, the concurrent detection of multiple conjugative plasmids in some isolates (e.g., C137A) raises concerns about the formation of “megaplasmids” or multiple conjugation events, which could give rise to strains resistant to an even broader spectrum of antimicrobial classes.

The findings of this study have important implications for public health and food safety management. First, identifying slaughterhouses as critical nodes for the coexistence and transmission of dominant clones and resistance plasmids provides a scientific foundation for targeted interventions. Enhancing biosecurity protocols, implementing rigorous sanitation and disinfection measures, and adopting risk-based microbiological monitoring at slaughter points are effective strategies to interrupt the transmission of resistant bacteria along the food chain. Second, the study highlights considerable uncertainty (with an average genotype-phenotype concordance of 61.7%) when relying solely on genotypic detection to predict phenotypic resistance. This underscores the necessity, in both public health surveillance and clinical diagnostics, of combining the traceability advantages offered by WGS with the phenotypic certainty of traditional antimicrobial susceptibility testing, thereby creating a complementary surveillance system. Finally, from a broader perspective, effectively curbing the spread of MDR *Salmonella* requires coordinated action within a One Health framework. This includes promoting prudent antimicrobial use at the farm level to reduce selective pressure; enforcing risk-based controls during food processing and distribution; and establishing an integrated genomic surveillance network that links veterinary, food regulatory, and public health sectors. Such coordination is essential for early detection and precise intervention against high-risk clones and MGEs.

We acknowledge certain limitations due to funding constraints. Although the genomic analysis of 19 representative isolates provided valuable insights into transmission dynamics, it may not fully capture the population’s genetic diversity. Additionally, the use of short-read sequencing limited comprehensive plasmid assembly. Future studies would benefit from incorporating long-read sequencing technologies to fully resolve plasmid structures, complemented by conjugation experiments to functionally validate plasmid transferability and stability.

## 5. Conclusions

In summary, the dissemination of MDR *Salmonella* within this system is a dual-track process. Clonal expansion of well-adapted lineages (ST1541, ST50) serves as a stable vehicle for resistance maintenance, while horizontal transfer of conjugative plasmids (IncI-gamma-K1, IncX1) facilitates rapid spread of resistance traits across isolate boundaries. Together, these mechanisms create a highly resilient and adaptable resistance network. Our findings highlight the need for integrated One Health interventions that both strengthen biosecurity at critical control points, such as slaughterhouses, to interrupt transmission and promote antimicrobial stewardship to reduce selective pressure. Additionally, establishing a coordinated genomic surveillance network is crucial for the early detection and containment of these high-risk clones and MGEs.

## Figures and Tables

**Table 1:** Genome sequencing and assembly statistics of 19 representative *S. enteritidis* isolates from the chicken production chain.

Sample ID	Source	Season	GC Content (%)	N50 (bp)	Completeness (%)	Genome Length (bp)	No of Contigs
C10A	Slaughterhouse	Rainy	52.12	256,957	100	4,968,700	61
C27A	Slaughterhouse	Rainy	52.18	528,820	100	4,903,582	44
C65A	Market	Rainy	51.98	456,352	99.71	4,992,687	52
C81A	Market	Rainy	52.05	452,366	99.71	5,087,732	51
C84A	Market	Rainy	51.95	321,932	100	4,879,865	334
C85A	Market	Rainy	52.25	329,450	100	4,881,272	41
C102A	Slaughterhouse	Summer	51.95	287,836	100	4,993,429	58
C137A	Market	Summer	52.12	239,014	100	4,742,180	96
C138A	Market	Summer	51.7	269,537	100	4,814,615	76
C149A	Slaughterhouse	Winter	52.13	390,398	100	4,761,950	70
C151A	Slaughterhouse	Winter	52.13	338,492	100	4,816,813	72
C152A	Slaughterhouse	Winter	52.12	239,014	100	5,089,386	94
C154A	Slaughterhouse	Winter	52.24	373,188	100	4,979,783	51
C188A	Slaughterhouse	Winter	52.02	307,925	99.97	5,088,165	54
C193A	Market	Winter	52.11	406,298	100	4,851,131	40
C195A	Market	Winter	52.02	307,925	99.97	4,847,405	56
C200A	Market	Winter	51.70	323,345	100	4,885,945	72
C205A	Market	Winter	52.12	183,935	100	5,013,442	81
C222A	Market	Winter	51.70	269,711	100	4,674,178	72

## Data Availability

The whole genome sequences generated in this study contain sensitive information that could potentially compromise the privacy of the participants. In accordance with the ethical approvals obtained for this study and the informed consent agreements that prioritize participant confidentiality, the raw sequence data are not deposited in public repositories. However, in the spirit of open science, all processed data (e.g., variant calls, association statistics) and analyses necessary to replicate the findings are available in the Supplementary Materials. For researchers who wish to access the raw data for specific scientific purposes under a data use agreement, inquiries can be directed to the corresponding author.

## Supplementary Materials

The following supporting information can be downloaded at: https://www.mdpi.com/article/10.3390/pathogens15010075/s1, Figure S1: Co-occurrence network analysis of plasmids and antibiotic resistance genes; Table S1: Profile of AMR, Plasmid, and Virulence Genes for the 19 Strains.
