# Supplementary material for: Clonal and Plasmid-Mediated Dissemination of Multidrug-Resistant Salmonella Enteritidis in Chicken Production, Northeastern Thailand
Source: Pathogens. 2026 Jan 10;15(1):75. doi: 10.3390/pathogens15010075 (PMC12844969; doi:10.3390/pathogens15010075)
Supplement: Supplementary file 1 [file pathogens-15-00075-s001.zip › pathogens-4043507-supplementary.pdf]

Supplementary data

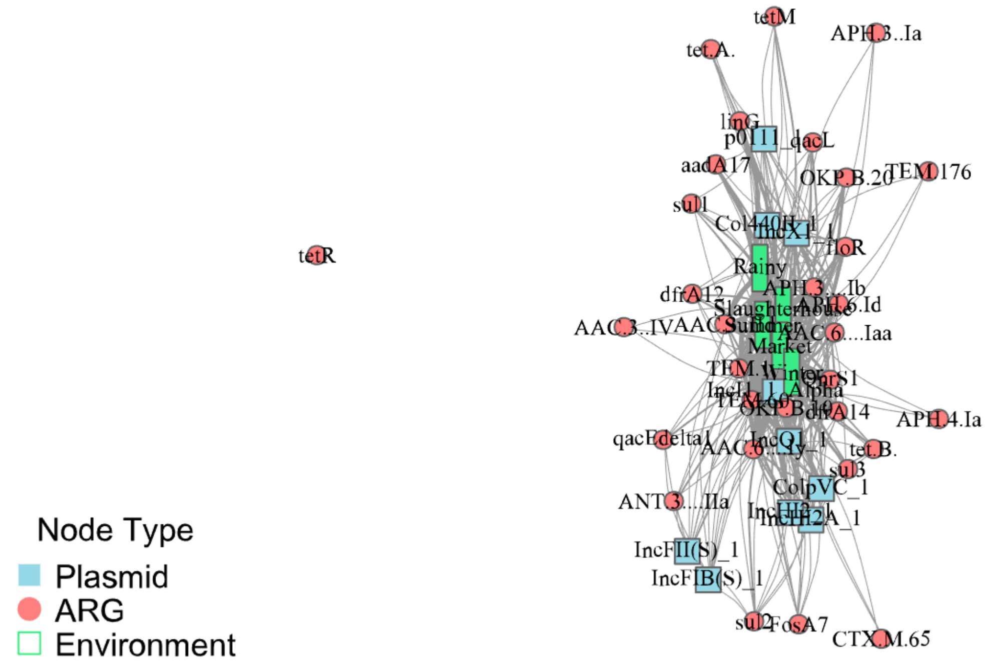

Figure S1 Co-occurrence network analysis of plasmids and antibiotic resistance genes

This network diagram illustrates 291 connections among 46 nodes (10 plasmid replicons, 33 antibiotic resistance genes, and 3 environmental factors). Node size reflects connectivity, while edge thickness indicates co-occurrence frequency. With a network density of 0.281, it exhibits moderately connected structural characteristics. Four major functional clusters were identified, with IncI1_1_Alpha, IncX1_1, and IncQ1_1 plasmids serving as core hub nodes that play crucial connecting roles within the network.

Table S1. Profile of AMR, Plasmid, and Virulence Genes for the 19 Strains

Sample ID	Antimicrobial resistance genes	Virulence genes	Main SPI	Plasmid	Antibiotic susceptibility testing pattern
C10A	*AAC(6')-Iy, OKP-B-10,gyrA , TEM-60*	*fimA,fimC,fimD,fimF,fimH,fimI,fimW,fimY,fimZ,invA,invB,invC,invE,invF,invG,invH,invI,invJ,lpfA,lpfB,lpfC,lpfD,lpfE,sipC,sipD,sitA,sitB,sitC,sitD,sopA,sopB,sopD,sopD2,sopE2,ssaC,ssaD,ssaE,ssaG,ssaH,ssaI,ssaJ,ssaK,ssaL,ssaM,ssaN,ssaO,ssaP,ssaQ,ssaR,ssaS,ssaT,ssaU,ssaV,sseA,sseB,sseC,sseD,sseE,sseF,sseG,sseJ,sseK1,sseK2,sseL*	T3SS(SPI-1/SPI-2)	-	Nor
C27A	*AAC(3)-IId, AAC(6')-Iy, ANT(3'')-IIa, OKP-B-10, TEM-1, TEM-60, qacL, sul3*	*fimA,fimB,fimC,fimD,fimF,fimH,fimI,fimW,fimY,fimZ,invA,invB,invC,invE,invF,invG,invH,invI,invJ,lpfA,lpfB,lpfC,lpfD,lpfE,sipA,sipB,sipC,sipD,sitA,sitB,sitC,sitD,sopA,sopB,sopD,sopD2,sopE2,ssaC,ssaD,ssaE,ssaG,ssaH,ssaI,ssaJ,ssaK,ssaL,ssaM,ss*	T3SS(SPI-1/SPI-2)	*IncI_gamma_K1*	S+GM+AM

		aN,ssaO,ssaP,ssaQ,ssaR,ssaS,ssaT,ssaU,ssaV,sseA,sseB,sseC,sseD,sseE,sseF,sseG,sseJ,sseK2,sseL			
C65A	*AAC(6')-Iy, APH(3')-Ia, OKP-B-20, QnrS1, TEM-176, TEM-60, dfrA14, floR, tet(A)*	*fimA,fimC,fimD,fimF,fimH,fimI,fimW,fimY,fimZ,invA,invB,invC,invE,invF,invG,invH,invI,invJ,lpfA,lpfB,lpfC,lpfD,lpfE,sipC,sipD,sitA,sitB,sitC,sitD,sopA,sopB,sopD,sopD2,sopE2,ssaC,ssaD,ssaE,ssaG,ssaH,ssaI,ssaJ,ssaK,ssaL,ssaM,ssaN,ssaO,ssaP,ssaQ,ssaR,ssaS,ssaT,ssaU,ssaV,sseA,sseB,sseC,sseD,sseE,sseF,sseG,sseI/srfH,sseJ,sseK1,sseK2,sseL*	T3SS(SPI-1/SPI-2)	*IncX1*	S+TE+GM+SXT+C+AM
C81A	*AAC(6')-Iaa, OKP-B-10, QnrS1*	*fimA,fimC,fimD,fimF,fimH,fimI,fimW,fimY,fimZ, inv,invA,invB,invC,invE,invF,invG,invH,invI,invJ,lpfA,lpfB,lpfC,lpfD,lpfE,sipC,sipD,sitA,sitB,sitC,sitD,sopA,sopB,sopD,sopD2,sopE2,ssaC,ssaD,ssaE,ssaG,ssaH,ssaI,ssaJ,ssaK,ssaL,ssaM,ssaN,ssaO,ssaP,ssaQ,ssaR,ssaS,ssaT,ssaU,ssaV,sseA,sseB,sseC,sseD,sseE,sseF,sseG,sseJ,sseK1,sseK2,sseL*	T3SS(SPI-1/SPI-2)	*Col_related*	
C84A	*AAC(6')-Iaa, OKP-B-20, QnrS1, TEM-60*	*fimA,fimC,fimD,fimF,fimH,fimI,fimW,fimY,fimZ,fljB,invA,invB,invC,invE,invF,invG,invH,invI,invJ,lpfA,lpfB,lpfC,lpfD,lpfE,sipC,sipD,sitA,sitB,sitC,sitD,sopA,sopB,sopD,sopD2,sopE2,ssaC,ssaD,ssaE,ssaG,ssaH,ssaI,ssaJ,ssaK,ssaL,ssaM,ssaN,ssaO,ssaP,ssaQ,ssaR,ssaS,ssaT,ssaU,ssaV,sseA,sseB,sseC,sseD,sseE,sseF,sseG,sseI/srfH,sseJ,sseK2,sseL*	T3SS(SPI-1/SPI-2)	*Col_related*	TE+GM+SXT+CTX+C+AM+CAZ+AM+CRO
C85A	*AAC(6')-Iy, OKP-B-10, TEM-60*	*fimA,fimC,fimD,fimF,fimH,fimI,fimW,fimY,fimZ,fljB,invA,invB,invC,invE,invF,invG,invH,invI,invJ,lpfA,lpfB,lpfC,lpfD,lpfE,sipC,sipD,sitA,sitB,sitC,sitD,sopA,sopB,sopD,sopD2,sopE2,ssaC,ssaD,ssaE,ssaG,ssaH,ssaI,ssaJ,ssaK,ssaL,ssaM,ssaN,ssaO,ssaP,ssaQ,ssaR,ssaS,ssaT,ssaU,ssaV,sseA,sseB,sseC,sseD,sseE,sseF,sseG,sseI/srfH,sseJ,sseK2,sseL*	T3SS(SPI-1/SPI-2)	-	S+CTX+C+AM+CAZ+CRO
C102A	*AAC(3)-IId, AAC(6')-Iaa, OKP-B-20, TEM-1, aadA17, linG*	*fimA,fimC,fimD,fimF,fimH,fimI,fimW,fimY,fimZ,invA,invB,invC,invE,invF,invG,invH,invI,invJ,lpfA,lpfB,lpfC,lpfD,lpfE,sipC,sipD,sitA,sitB,sitC,sitD,sopA,sopB,sopD,sopD2,sopE2,ssaC,ssaD,ssaE,ssaG,ssaH,ssaI,ssaJ,ssaK,ssaL,ssaM,ssaN,ssaO,ssaP,ssaQ,ssaR,ssaS,ssaT,ssaU,ssaV,sseA,sseB,sseC,sseD,sseE,sseF,sseG,sseI/srfH,sseJ,sseK1,sseK2,sseL*	T3SS(SPI-1/SPI-2)	*IncHI1B*	S+TE+AM+CAZ+CRO
C137A	*AAC(3)-IId, AAC(6')-Iaa, APH(3'')-Ib, APH(6)-Id, OKP-B-10, TEM-1, aadA17, linG, sul2, tet(B), tetR*	*fimA,fimB,fimC,fimD,fimF,fimH,fimI,fimW,fimY,fimZ,fljB,invA,invB,invC,invE,invF,invG,invH,invI,invJ,lpfA,lpfB,lpfC,lpfD,lpfE,sipC,sipD,sitA,sitB,sitC,sitD,sopA,sopB,sopD,sopD2,sopE2,ssaC,ssaD,ssaE,ssaG,ssaH,ssaI,ssaJ,ssaK,ssaL,ssaM,ssaN,ssaO,ssaP,ssaQ,ssaR,ssaS,ssaT,ssaU,ssaV,sseA,sseB,sseC,sseD,sseE,sseF,sseG,sseJ,sseK1,sseL*	T3SS(SPI-1/SPI-2)	*IncQ1*	S+TE+GM+NA+SXT+AM
C138A	*AAC(3)-IId, APH(3'')-Ib, APH(6)-Id, OKP-B-10, QnrS1, TEM-1, dfrA12, floR, sul1, tet(A), tet(M)*	*fimA,fimC,fimD,fimF,fimH,fimI,fimW,fimY,fimZ, fliC,fljB,inv,invA,invB,invC,invE,invF,invG,invH,invI,invJ,lpfA,lpfB,lpfC,lpfD,lpfE,sipC,sipD,sitA,sitB,sitC,sitD,sopA,sopB,sopD,sopD2,sopE2,ssaC,ssaD,ssaE,ssaG,ssaH,ssaI,ssaJ,ssaK,ss*	T3SS(SPI-1/SPI-2)	*IncHI1B/IncHI2A, IncQ1*	S+TE+GM+NA+SXT+CTX+AM

		aL,ssaM,ssaN,ssaO,ssaP,ssaQ,ssaR,ssaS,ssaT,ssaU,ssaV,sseA,sseB,sseC,sseD,sseE,sseF,sseG,sseJ,sseK1,sseL			
C149A	*AAC(3)-IId, AAC(6')-Iy, OKP-B-10, TEM-1, TEM-60, aadA17, linG*	*fimA,fimB,fimC,fimD,fimF,fimH,fimI,fimW,fimY,fimZ,fljB,invA,invB,invC,invE,invF,invG,invH,invI,invJ,lpfA,lpfB,lpfC,lpfD,lpfE,sipC,sipD,sitA,sitB,sitC,sitD,sopA,sopB,sopD,sopD2,sopE,sopE2,spvB,spvC,spvR,ssaC,ssaD,ssaE,ssaG,ssaH,ssaI,ssaJ,ssaK,ssaL,ssaM,ssaN,ssaO,ssaP,ssaQ,ssaR,ssaS,ssaT,ssaU,ssaV,sseA,sseB,sseC,sseD,sseE,sseF,sseG,sseI/srfH,sseJ,sseK1,sseK2,sseL*	T3SS(SPI-1/SPI-2)	*IncI_gamma_K1*	S+TE+GM+NA+AM+AMC
C151A	*AAC(3)-IId, AAC(6')-Iy, OKP-B-10, TEM-1, TEM-60, aadA17, linG*	*fimA,fimB,fimC,fimD,fimF,fimH,fimI,fimW,fimY,fimZ,fljB,invA,invB,invC,invE,invF,invG,invH,invI,invJ,lpfA,lpfB,lpfC,lpfD,lpfE,sipC,sipD,sitA,sitB,sitC,sitD,sopA,sopB,sopD,sopD2,sopE2,ssaC,ssaD,ssaE,ssaG,ssaH,ssaI,ssaJ,ssaK,ssaL,ssaM,ssaN,ssaO,ssaP,ssaQ,ssaR,ssaS,ssaT,ssaU,ssaV,sseA,sseB,sseC,sseD,sseE,sseF,sseG,sseJ,sseK1,sseK2,sseL*	T3SS(SPI-1/SPI-2)	*IncI_gamma_K1*	S+AM
C152A	*AAC(3)-IId, AAC(6')-Iaa, APH(3'')-Ib, APH(6)-Id, OKP-B-10, TEM-1, TEM-60, aadA17, linG, sul2, tet(B), tetR*	*fimA,fimB,fimC,fimD,fimF,fimH,fimI,fimW,fimY,fimZ,fliC,invA,invB,invC,invE,invF,invG,invH,invI,invJ,lpfA,lpfB,lpfC,lpfD,lpfE,sipC,sipD,sitA,sitB,sitC,sitD,sopA,sopB,sopD,sopD2,sopE2,ssaC,ssaD,ssaE,ssaG,ssaH,ssaI,ssaJ,ssaK,ssaL,ssaM,ssaN,ssaO,ssaP,ssaQ,ssaR,ssaS,ssaT,ssaU,ssaV,sseA,sseB,sseC,sseD,sseE,sseF,sseG,sseJ,sseK1,sseK2,sseL*	T3SS(SPI-1/SPI-2)	*IncI_gamma_K1, IncQ1*	S+TE+GM+NA+AM+AMC
C154A	*AAC(3)-IId, AAC(6')-Iy, OKP-B-10, TEM-1, TEM-60*	*fimA,fimC,fimD,fimF,fimH,fimI,fimW,fimY,fimZ,fljB,invA,invB,invC,invE,invF,invG,invH,invI,invJ,lpfA,lpfB,lpfC,lpfD,lpfE,sipC,sipD,sitA,sitB,sitC,sitD,sopA,sopB,sopD,sopD2,sopE2,ssaC,ssaD,ssaE,ssaG,ssaH,ssaI,ssaJ,ssaK,ssaL,ssaM,ssaN,ssaO,ssaP,ssaQ,ssaR,ssaS,ssaT,ssaU,ssaV,sseA,sseB,sseC,sseD,sseE,sseF,sseG,sseJ,sseK1,sseK2,sseL*	T3SS(SPI-1/SPI-2)	*IncI_gamma_K1*	S+TE+GM+NA+AM
C188A	*AAC(6')-Iy, APH(3'')-Ib, APH(6)-Id, FosA7, OKP-B-10, TEM-1, TEM-60, dfrA14, sul3, tet(A)*	*fimA,fimC,fimD,fimF,fimH,fimI,fimW,fimY,fimZ,invA,invB,invC,invE,invF,invG,invH,invI,invJ,lpfA,lpfB,lpfC,lpfD,lpfE,sipC,sipD,sitA,sitB,sitC,sitD,sopA,sopB,sopD,sopD2,sopE2,ssaC,ssaD,ssaE,ssaG,ssaH,ssaI,ssaJ,ssaK,ssaL,ssaM,ssaN,ssaO,ssaP,ssaQ,ssaR,ssaS,ssaT,ssaU,ssaV,sseA,sseB,sseC,sseD,sseE,sseF,sseG,sseJ,sseK1,sseK2,sseL*	T3SS(SPI-1/SPI-2)	*IncX1*	S+TE+GM+NA+SXT+AM+AMC
C193A	*AAC(6')-Iy, OKP-B-10, gyrA*	*fimA,fimC,fimD,fimF,fimH,fimI,fimW,fimY,fimZ,fljB,invA,invB,invC,invE,invF,invG,invH,invI,invJ,lpfA,lpfB,lpfC,lpfD,lpfE,sipC,sipD,sitA,sitB,sitC,sitD,sopA,sopB,sopD,sopD2,sopE2,ssaC,ssaD,ssaE,ssaG,ssaH,ssaI,ssaJ,ssaK,ssaL,ssaM,ssaN,ssaO,ssaP,ssaQ,ssaR,ssaS,ssaT,ssaU,ssaV,sseA,sseB,sseC,sseD,sseE,sseF,sseG,sseJ,sseK2,sseL*	T3SS(SPI-1/SPI-2)	*IncFIB - FII*	AM+AMC
C195A	*AAC(6')-Iy, APH(3'')-Ib, APH(6)-Id, FosA7, OKP-B-20, QnrS1, TEM-1, TEM-60, dfrA14, sul3, tet(A)*	*fimA,fimC,fimD,fimF,fimH,fimI,fimW,fimY,fimZ,fljB,invA,invB,invC,invE,invF,invG,invH,invI,invJ,lpfA,lpfB,lpfC,lpfD,lpfE,sipC,sipD,sitA,sitB,sitC,sitD,sopA,sopB,sopD,sopD2,sopE2,ssaC,ssaD,ssaE,ssaG,ssaH,ssaI,ssaJ,ssaK,ssaL,ssaM,ssaN,ssaO,ssa*	T3SS(SPI-1/SPI-2)	-	S+TE+GM+NA+SXT+C+AM

		P,ssaQ,ssaR,ssaS,ssaT,ssaU,ssaV,sseA,sseB,sseC,sseD,sseE,sseF,sseG,sseJ,sseK1,sseL			
C200A	*AAC(3)-IId, APH(3'')-Ib, APH(6)-Id, OKP-B-10, QnrS1, TEM-1, dfrA12, floR, sul1, tet(A), tet(M)*	*fimA,fimC,fimD,fimF,fimH,fimI,fimW,fimY,fimZ,fliC,inv,invA,invB,invC,invE,invF,invG,invH,invI,invJ,lpfA,lpfB,lpfC,lpfD,lpfE,sipC,sipD,sitA,sitB,sitC,sitD,sopA,sopB,sopD,sopD2,sopE2,ssaC,ssaD,ssaE,ssaG,ssaH,ssaI,ssaJ,ssaK,ssaL,ssaM,ssaN,ssaO,ssaP,ssaQ,ssaR,ssaS,ssaT,ssaU,ssaV,sseA,sseB,sseC,sseD,sseE,sseF,sseG,sseJ,sseK1,sseK2,sseL*	T3SS(SPI-1/SPI-2)	*IncQ1*	S+TE+GM+NA+SXT+CTX+AM
C205A	*AAC(3)-IV, AAC(6')-Iy, ANT(3'')-IIa, APH(4)-Ia, CTX-M-65, OKP-B-10, gyrA , TEM-60, dfrA14, floR, qacEdelta1, sul1, tet(A)*	*fimA,fimC,fimD,fimF,fimH,fimI,fimW,fimY,fimZ,fljB,invA,invB,invC,invE,invF,invG,invH,invI,invJ,lpfA,lpfB,lpfC,lpfD,lpfE,sipC,sipD,sitA,sitB,sitC,sitD,sopA,sopB,sopD,sopD2,sopE2,ssaC,ssaD,ssaE,ssaG,ssaH,ssaI,ssaJ,ssaK,ssaL,ssaM,ssaN,ssaO,ssaP,ssaQ,ssaR,ssaS,ssaT,ssaU,ssaV,sseA,sseB,sseC,sseD,sseE,sseF,sseG,sseJ,sseK1,sseK2,sseL*	T3SS(SPI-1/SPI-2)	*IncFIB*	S+TE+GM+NA+SXT+IPM+CTX+CIP+AM
C222A	*AAC(3)-IId, AAC(6')-Iaa, APH(3'')-Ib, APH(6)-Id, OKP-B-10, QnrS1, TEM-1, dfrA12, floR, sul1, tet(A), tet(M)*	*fimA,fimC,fimD,fimF,fimH,fimI,fimW,fimY,fimZ,fliC,inv,invA,invB,invC,invE,invF,invG,invH,invI,invJ,lpfA,lpfB,lpfC,lpfD,lpfE,sipC,sipD,sitA,sitB,sitC,sitD,sopA,sopB,sopD,sopD2,sopE2,ssaC,ssaD,ssaE,ssaG,ssaH,ssaI,ssaJ,ssaK,ssaL,ssaM,ssaN,ssaO,ssaP,ssaQ,ssaR,ssaS,ssaT,ssaU,ssaV,sseA,sseB,sseC,sseD,sseE,sseF,sseG,sseJ,sseK1,sseK2,sseL*	T3SS(SPI-1/SPI-2)	*IncHI1B/IncHI2A, IncQ1*	S+TE+GM+NA+SXT+IPM+AM
